# Radium-223 Treatment Produces Prolonged Suppression of Resident Osteoblasts and Decreased Bone Mineral Density in Trabecular Bone in Osteoblast Reporter Mice

**DOI:** 10.3390/cancers16142603

**Published:** 2024-07-21

**Authors:** Song-Chang Lin, Guoyu Yu, Paul G. Corn, Jossana Damasco, Yu-Chen Lee, Jian H. Song, Nora M. Navone, Christopher J. Logothetis, Marites P. Melancon, Theocharis Panaretakis, Sue-Hwa Lin

**Affiliations:** 1 Department of Translational Molecular Pathology, MD Anderson Cancer Center, University of Texas, Houston, TX 77030, USA; sc8619l@gmail.com (S.-C.L.); gyu1@mdanderson.org (G.Y.); yuchencheng@hotmail.com (Y.-C.L.); 2 Department of Genitourinary Medical Oncology, MD Anderson Cancer Center, University of Texas, Houston, TX 77030, USA; pcorn@mdanderson.org (P.G.C.); jhsong@mdanderson.org (J.H.S.); nnavone@mdanderson.org (N.M.N.); clogothe@mdanderson.org (C.J.L.); 3 Department of Interventional Radiology, MD Anderson Cancer Center, University of Texas, Houston, TX 77030, USA; damascoja@gmail.com (J.D.); mmelancon@mdanderson.org (M.P.M.); 4 UTHealth Houston Graduate School of Biomedical Sciences, MD Anderson Cancer Center, University of Texas, Houston, TX 77030, USA

**Keywords:** Radium-223, osteoblast reporter mice, osteoblasts, bone mass, prostate cancer

## Abstract

**Simple Summary:**

Radium 223 (Ra-223) is a radiopharmaceutical that targets tumor-induced osteoblasts (bone-forming cells). Ra-223 reduces bone pain and prolongs overall survival in men with bone-metastatic, castrate-resistant prostate cancer. However, increased fracture risk in skeletal sites with no bone metastasis has been observed in patients treated with Ra-223. The aim of this study was to examine the effects of Ra-223 on resident osteoblasts and normal bone structure in mouse models. Upon Ra-223 treatment, 70% of resident osteoblasts were reduced within 2 days, and the reduction lasted for at least 18 weeks. Ra-223 reduced the osteoblasts mainly localized in trabecular bone areas. Ra-223 also reduced bone mineral density and altered bone microstructure in the trabecular area of femurs. Furthermore, Ra-223 treatment also significantly reduced tumor-induced osteoblasts. These studies show that Ra-223 affects the structure of bones that are not involved in bone metastasis. Strategies that improve bone health may reduce fracture risk in patients receiving Ra-223.

**Abstract:**

Radium 223 (Ra-223) is an α-emitting bone-homing radiopharmaceutical that targets tumor-induced osteoblasts and is used to reduce bone pain and prolong overall survival in men with bone-metastatic, castrate-resistant prostate cancer. However, increased fracture risk in skeletal sites with no bone metastasis has been observed in patients treated with Ra-223. Both luciferase- or green fluorescence protein (GFP)-labeled osteoblast reporter mice were used to monitor the effect of Ra-223 on resident osteoblasts and normal bone structure. Upon Ra-223 treatment, 70% of resident osteoblasts were reduced within 2 days, and the osteoblast reduction lasted for at least 18 weeks without detectable recovery, as measured by in vivo bioluminescent imaging. In GFP-labeled osteoblast reporter mice, Ra-223 mainly reduced osteoblasts localized in the trabecular bone areas; the osteoblasts in the growth plates were less affected. Micro-computed tomography analyses showed that Ra-223 significantly reduced bone mineral density and bone microstructure in the trabecular area of femurs but not in the cortical bone. Tumor-induced bone was generated by inoculating osteogenic TRAMP-BMP4 prostate cancer cells into the mouse femurs; Ra-223 treatment significantly reduced tumor-induced osteoblasts. Our study shows that Ra-223 affects bone structures that are not involved in bone metastasis. Strategies that improve bone health may reduce fracture risk in patients receiving Ra-223.

## 1. Introduction

Prostate cancer metastasizes primarily to bone [1]. The development of bone metastases in castration-resistant prostate cancer (bmCRPC) signifies the lethal progression of the disease. The incidence of bone involvement in patients with metastatic disease is greater than 85%, and bone lesions can cause significant morbidity, including pain and skeletal-related complications. BmCRPC exhibits a unique bone-forming phenotype that dominates the clinical presentation of treatment-refractory disease [1]. It has been shown that tumor-induced aberrant bone formation enhanced the survival and progression of prostate cancer [2,3,4,5,6]. Several studies have indicated a role of osteoblasts in prostate cancer cell proliferation [7,8], migration [3], and therapy resistance [9]. This body of work has provided the rationale for blocking tumor-induced bone formation as a strategy in controlling prostate cancer growth in bone to reduce morbidity and mortality in advanced disease. 

Bone-seeking radiopharmaceuticals are calcium mimetics that deliver ionizing radiation specifically to targeted areas of increased osteoblastic activity. β-emitters, including strontium 89 dichloride [10,11] and samarium 153 lexidronam (^153^Sm-EDTMP) [12,13,14], have been used to treat bone metastasis. However, the relatively long particle track length and low linear energy transfer (LET) of β-emitters result in bone marrow suppression, which limits their usefulness. In contrast, radium 223 (Ra-223) is an α-emitting radiopharmaceutical that has a relatively short particle track length and high LET, which can potently induce DNA damage and apoptosis in adjacent cells [15]. Ra-223 has been shown to reduce bone pain and prolong overall survival in men with bmCRPC [16]. In 2013, Ra-223 (Xofigo) became the first α-emitter approved by the US Food and Drug Administration for symptomatic bmCRPC. 

Although Ra-223 significantly improves overall survival, prostate-specific antigen (PSA) responses are rare and not associated with clinical benefit, while reductions in alkaline phosphatase (a marker of osteoblast activity) are associated with better survival [16]. These data support the importance of tumor-induced bone in disease lethality and as a valid therapy target. However, an increased risk of fracture was observed with Ra-223 treatment [17]. Because Ra-223 principally targets osteoblasts, the idea of combining Ra-223 with agents that more potently target tumor cells has been pursued to improve treatment efficacy. However, the combination of Ra-223 with abiraterone, a second-generation androgen-receptor inhibitor, resulted in an increased bone fracture rate and no survival advantage in patients with bmCRPC [18]. Interestingly, the analysis of bone fractures in this combination trial (ERA 223) suggested that fractures in the Ra-223 group occurred at skeletal sites with no bone metastasis, mainly due to osteoporotic fractures [18]. These observations raise questions about the effect of Ra-223 on bone health in non-tumor-affected areas.

Understanding the effects of Ra-223 on non-tumor-affected bone is necessary for optimizing the use of Ra-223, especially in combination with other therapies. The effect of Ra-223 on bone metastatic prostate cancer regarding therapeutic efficacy as well as mechanisms of action and subsequent resistance have been extensively studied [19,20,21]. However, the knowledge on the effects of Ra-223 on resident osteoblasts in normal bone regeneration and bone quality is limited. Because bone fractures in non-tumor-affected areas are frequently observed in patients undergoing Ra-223 treatment [18], we hypothesized that Ra-223 affects the quality of normal bone. In this study, two transgenic mouse models with osteoblast-specific expression of luciferase or GFP were used to examine the effects of Ra-223 on resident osteoblasts and normal bone structures to provide insights into the effects on the bone health of patients undergoing Ra-223 treatments. 

## 2. Materials and Methods

### 2.1. Animals

The collagen 1α1-luciferase transgenic mice with SCID immunodeficient background (Col-Luc/SCID) were generated as described previously [22]. Col-Luc/B6 mice were generated by crossing Col-Luc/SCID mice with C57BL/6J mice (B6; Jackson Laboratory (Bar Harbor, ME), stock #000664) to generate heterozygotes (generation F1), which were then bred with C57BL/6J mice to generate Col-Luc/B6 mice. Transgenic Col-GFP/B6 mice were purchased from Jackson Laboratory (stock #013134). All animal studies were approved by the MD Anderson Institutional Animal Care and Use Committee. 

### 2.2. Treatment of Mice with Ra-223 and Bioluminescence Measurement

Mice were injected with Ra-223 dichloride (300 kilobecquerel (kBq)/kilogram (kg)) in 100 µL saline per mouse) via tail vein injection. The littermates or age-matched mice were used for comparison. Bioluminescence of luciferase activity (BLI) was measured using an IVIS 200 imaging system before Ra-223 treatment (day 0) and at day 2, week 1, and every 1–2 weeks after Ra-223 treatment. For measuring luciferase activity, mice were injected with 100 µL of D-luciferin solution (5 mg/mL) via i.p. The duration of each BLI measurement depends on the signal and either 30 s or an auto duration mode was used.

### 2.3. µCT Analysis and GFP Quantification

Col-GFP/B6 mice that were treated with or without Ra-223 were euthanized at designated time points, and the femurs were collected. After being fixed in 4% PFA, the femurs were analyzed by micro-computed tomography (μCT) using a Bruker SkyScan Micro CT 1276 scanner, with scanning at 13 µm resolution. The regions of interest (ROIs) for trabecular (Tb) and cortical (Cort) bone areas were identified and bone histomorphometry parameters, including bone mineral density (BMD), bone specific volume (BV/TV), bone specific surface (BS/TV), number of trabeculae (Tb number), and trabecular bone spacing (Tb separation), were determined. Subsequently, the femurs were decalcified, cryoprotected in sucrose gradients, and embedded in optimal cutting temperature (OCT) compound. The femurs were cut into 5 µm thick sections. The sections were GFP positive (endogenous), and the nuclei were stained with 4′,6-diamidino-2-phenylindole (DAPI). ImageJ software (version 1.51) was used to quantify the GFP levels of osteoblasts. The adjacent slide to this section was stained with hematoxylin and eosin (H&E) for histologic analysis. 

### 2.4. Generation of TRAMP-BMP4 Cell Line 

TRAMP-C2 cells were purchased from ATCC (Gaithersburg, MD) (CRL-2731, lot #63361790) passage 16 in 2016 and maintained in Dulbecco’s modified Eagle’s medium with 4 mM L-glutamine, adjusted to contain 1.5 g/L sodium bicarbonate, and 4.5 g/L glucose supplemented with 0.005 mg/mL bovine insulin and 10 nM dehydroisoandrosterone (90%), 5% FBS, 5% Nu Serum IV, and Penicillin/Streptomycin [23]. The TRAMP-BMP4 cell line was generated by using a bicistronic retroviral vector that contained mouse BMP4 (BMP4) cDNA and floxed-GFP through IRES. The primer sequences for constructing the pBMN-loxp-GFP plasmid were 5′-CCATGGATAACTTCGTATAGCATACATTATACGAAGTTATATGGTGAGCAAGGGCGAGGA-3′ and 5′-GTCGACATAACTTCGTATA ATGTATGC TATACGAAGTTAT TCACTTGTACAGCTCGTCCATGCC-3′. The cDNA encoding mouse bone morphogenetic protein (BMP4) was then inserted into bicistronic retroviral vector pBMN-loxp-GFP to generate pBMN-BMP4-IRES-loxp-GFP. TRAMP-C2 cells were transduced with retrovirus generated from pBMN-BMP4-IRES-loxp-GFP, and cells expressing BMP4 were selected through GFP sorting. The TRAMP-BMP4-GFP cells were infected with adenovirus-Crepase (Ad-Cre) (Vector Biolabs, Malvern, PA) to delete the GFP gene that was flanked by loxP sites. The top 5% of GFP-negative cells were collected through fluorescence-activated cell sorting as TRAMP-BMP4 cells. RT-PCR was used for BMP4 expression in TRAMP-C2 cells, using BMP4 primers (5′-GGATCCATGATTCCTGGTAACCGAATGCTG-3′ and 5′-GAATTCTCAATGATGATGATGATGATGATGATGTCAGCG GCA TCCACACCCCTCT-3′). Cell lines were confirmed to be free of mycoplasma using a MycoAlert Mycoplasma Detection kit (Lonza Bioscience, Houston, TX) (LT07-418).

### 2.5. Mice with TRAMP-BMP4 Tumors 

TRAMP-BMP4 cells (5 μL of 1 × 10^6^ cells/mL) were injected into the right femurs of Col-GFP mice (8–10 weeks old). Four weeks after implantation, mice were divided into two groups; one was treated with Ra-223 at 300 kBq/kg through tail vein injection, and the other was used as the control. Two weeks later, both femurs of each mouse were collected and fixed in 4% PFA at 4 °C. Femurs were decalcified in 0.25 M EDTA at 4 °C for 7 days with daily refreshing, equilibrated in 30% sucrose in PBS, and embedded in OCT compound. Femurs were sectioned at 5 μm and used for intrinsic GFP detection. 

### 2.6. Statistical Analysis

Data were expressed as the mean ± S.E.M. Student’s *t*-test was used for statistical analysis. 

Because littermates or age-matched mice were used to control the variables from mouse age and genetic background, the number of mice in each experimental group is limited by the size of the litter. In some experiments, two littermates of the same birth date were pooled. In some experiments, 4 mice per group was used. An effect size calculation was made. With a sample size of 4 mice per group, an effect size of 2.0 or 2.38 using a sample t-test of 1- or 2-sided 5% significance level, respectively, will have 80% power with 5% significance level. The power calculations were performed using G*power 3.1.9.7 [24].

## 3. Results

### 3.1. Ra-223 Treatment Decreases Tail Bone Luciferase Activity in a Col-Luc Osteoblast Reporter Mice

**Figure 1:**
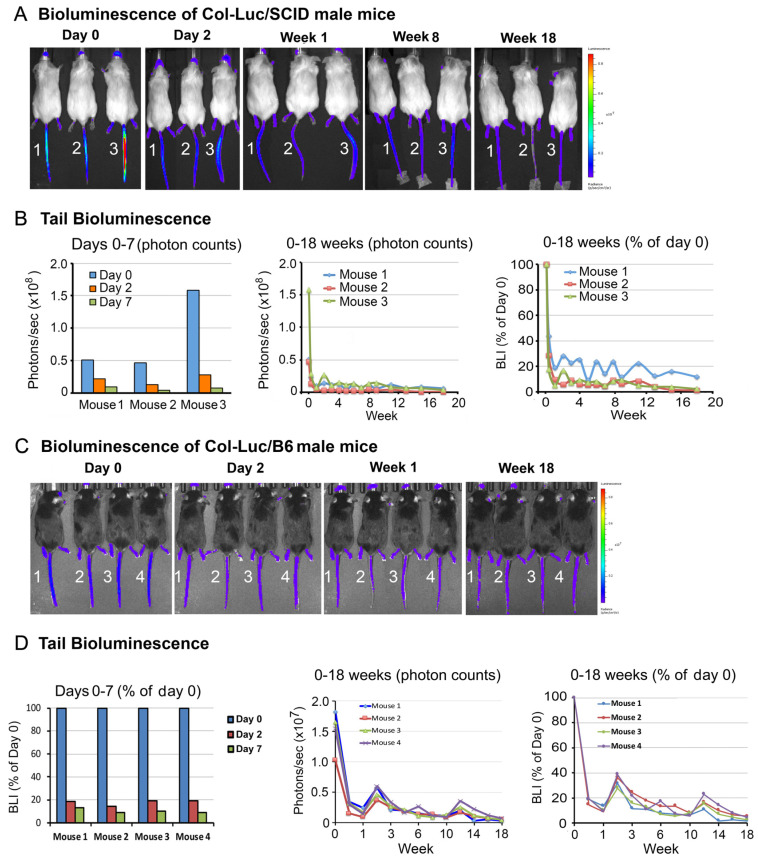
Ra-223 treatment decreases tail bone luciferase activity in male Col-Luc osteoblast reporter mice. (**A**,**B**) Col-Luc/SCID mice were treated with 300 kBq/kg Ra-223, and the luciferase activity was monitored for 18 weeks using an IVIS 200 imaging system. BLI was monitored right before the injection (day 0) and at the subsequent time points indicated. (**A**) Bioluminescence imaging (BLI) of three Col-Luc/SCID mice. (**B**) Tail BLI. Left panel, photon counts on days 0, 2, and 7. There was a fast reduction of BLI on day 2 following the treatment. Middle panel, photon counts from 0 to 18 weeks. Right panel, BLI percentage when compared to day 0. (**C**,**D**) Results for Col-Luc/B6 mice treated similarly as those shown in panels A and B.

To monitor osteoblast response to Ra-223, a luciferase-expressing osteoblast reporter mouse line (Col-Luc mouse) was used. This transgenic mouse line carries 2.3-kb Pro-α1(I) collagen promoter-driven luciferase transgene [25]. The proximal 2.3-kb Col1α1 promoter was shown to direct osteoblast-specific expression of luciferase protein in the transgenic mice [25]. This construct allows changes in the levels of osteoblasts in vivo to be monitored by luciferase activity. In previous studies, Col-Luc mice in a SCID background (Col-Luc/SCID) were generated and the luciferase expression was detected in the skull, femurs, tibias, and tail of newborn mice [22]. In adult Col-Luc/SCID mice, luciferase activity was mainly detected in the tail and feet (Figure 1A), likely due to the luciferase signal from osteoblasts being masked in other anatomic locations by the amount of overlying tissue. Nevertheless, these reporter mice allow us to longitudinally monitor osteoblast activity during Ra-223 treatment.

To examine the effects of duration of Ra-223 treatment on osteoblasts in vivo, Col-Luc/SCID male mice were injected intravenously via the tail vein with 300 kBq/kg of Ra-223, a dose previously used in a mouse model of prostate cancer bone metastasis [21], and monitored by bioluminescence imaging (BLI). Ra-223 treatment led to a 70% decrease in luciferase activity in the tails as early as on day 2 in a majority of mice (Figure 1A,B). On day 7, the luciferase activity was 11.1 ± 7.2% of initial activity (Figure 1B). Although the half-life of Ra-223 is 11.4 days, the luciferase activity remained low for the entire 18 weeks of the study period (Figure 1A,B). Similar results were observed in the bones in mouse feet (Figure S1). These observations indicate that a single Ra-223 dose has long-lasting effects on osteoblasts in normal mouse bones in SCID mice. 

Because Col-Luc/SCID mice have very low fertility rates and are immunodeficient, Col-Luc/SCID mice were crossed with C57BL/6 (B6) mice to generate Col-Luc/B6 mice. The effects of Ra-223 on osteoblasts in both sexes were then examined. When treated with 300 kBq/kg of Ra-223, both male (Figure 1C,D) and female Col-Luc/B6 (Figure S2) mice exhibited long-term (18 weeks) osteoblast reduction, similar to that observed in Col-Luc/SCID male mice (Figure 1A,B). These observations indicate that one dose of Ra-223 treatment reduced the resident osteoblasts to 10–20% of original levels for at least 18 weeks in both Col-Luc/SCID and Col-Luc/B6 mice. Interestingly, while regeneration of osteoblasts subsequent to Ra-223 decay was expected, recovery of luciferase activity from Ra-223 treatment was not observed for the duration of the 18-week follow-up time. 

### 3.2. Ra-223 Decreases GFP-Labeled Osteoblasts in Col-GFP Mice

**Figure 2:**
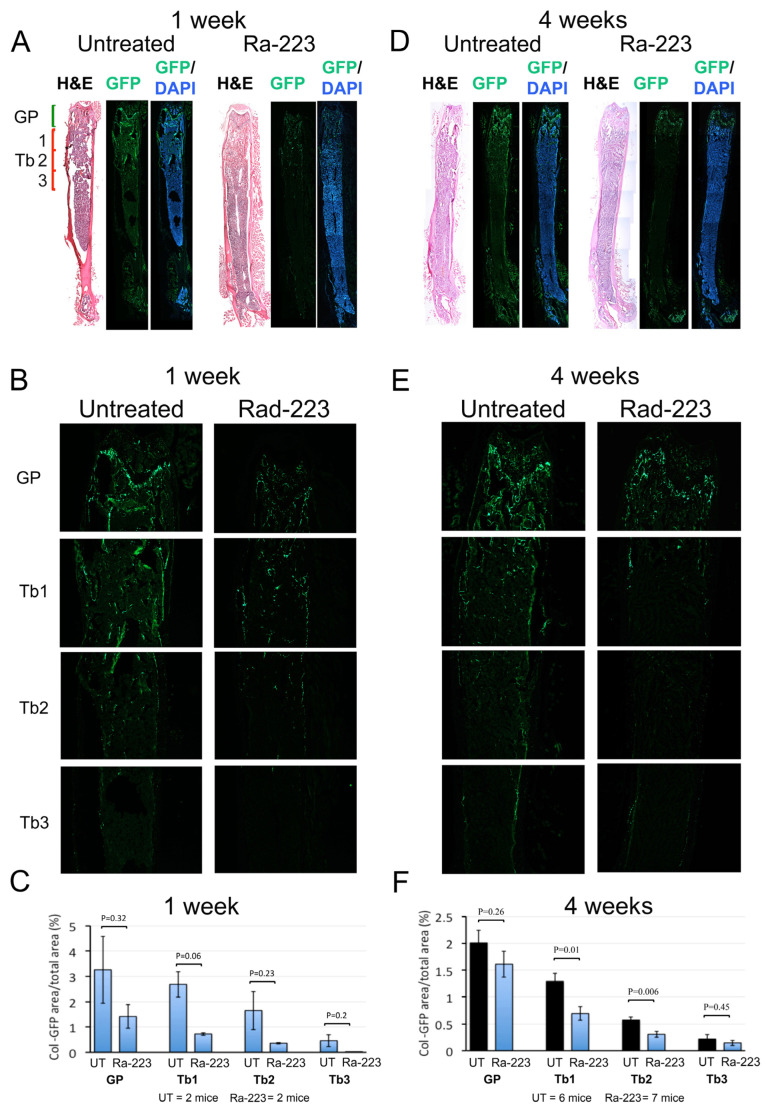
Ra-223 decreases GFP-labeled osteoblasts in Col-GFP/B6 osteoblast reporter mice at 1 and 4 weeks after treatment. Male Col-GFP/B6 mice (10 weeks old) were treated with one dose of Ra-223 at 300 kBq/kg. Age-matched Col-GFP/B6 mice were used as controls (untreated, UT). Frozen sections of both groups were imaged. (**A**) Representative images of whole femurs 1 week after Ra-223 treatment. (**B**) The growth plate (GP) and trabeculae (Tb) regions. (**C**) Col-GFP signals were quantified using ImageJ software. Although Ra-223 treatment showed a trend towards reduction in the number of GFP-labeled osteoblasts at 1 week after treatment, it did not reach statistical significance due to low sample size (*n* = 2 per group). (**D**–**F**) The same measures shown in panels A–C were repeated 4 weeks after Ra-223 treatment. (**D**) Representative images of whole femurs 4 weeks after Ra-223 treatment. (**E**) The decrease in osteoblast number was mainly observed in the metaphysis of the femur, an area of active trabecular bone formation. (**F**) Quantification of GFP levels by ImageJ software. With a sample size of 6 for untreated (UT) and 7 for Ra-223 treated, the effect sizes were 0.66, 1.67, 1.89, and 0.43 for GP, Tb1, Tb2, and Tb3, respectively. The powers of the test for GP, Tb1, Tb2, and Tb3 were 0.30, 0.88, 0.94, and 0.18, respectively, using a sample *t*-test (1-sided) and 5% significance level. These results showed that Ra-223 significantly inhibited osteoblasts present in trabeculae (Tb1 and Tb2) but not in the growth plate region (GP) in male mice 4 weeks after Ra-223 treatment.

**Figure 3:**
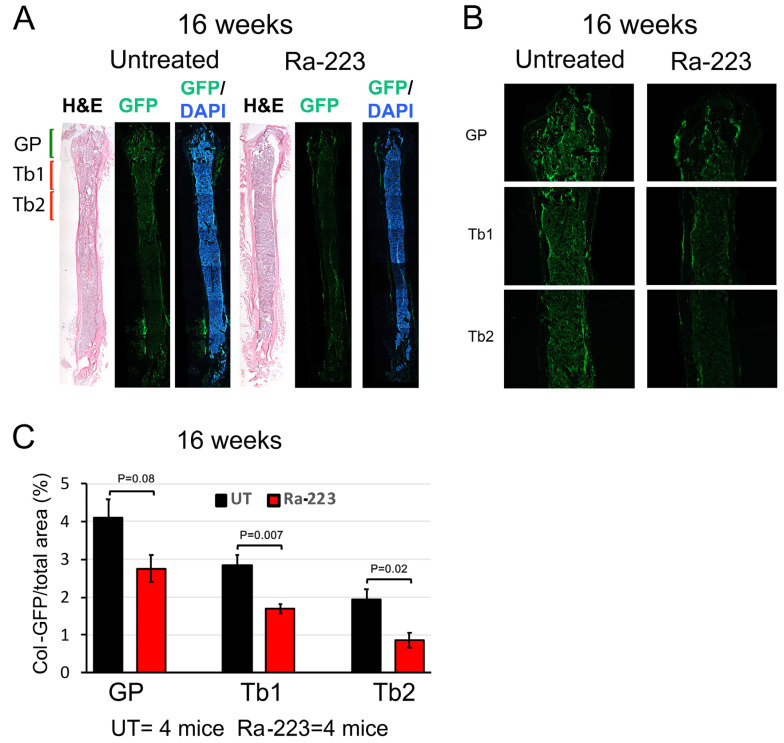
Ra-223 decreases GFP-labeled osteoblasts in Col-GFP/B6 osteoblast reporter mice at 16 weeks after treatment. The same measurements shown in Figure 2 were repeated 16 weeks after Ra-223 treatment. (**A**) Representative images of whole femurs 16 weeks after Ra-223 treatment. (**B**) The decrease in osteoblast number was mainly observed in the metaphysis of the femur, an area of active trabecular bone formation. (**C**) Quantification of GFP levels by ImageJ software. With a sample size of 4 mice for untreated (UT) and 4 mice in Ra-223 treated group, the effect sizes were 1.84, 3.60, and 3.25 for GP, Tb1, and Tb2, respectively. The powers of the test for GP, Tb1, and Tb2 were 0.74, 1.00, and 0.99, respectively, using a sample *t*-test (1-sided) and 5% significance level. These results showed that Ra-223 significantly inhibited osteoblasts present in the Tb1 and Tb2 regions in male mice 16 weeks after Ra-223 treatment.

Although Col-Luc mice allowed for the monitoring of the osteoblast response to Ra-223 in vivo, luciferase activity is unsuitable for localization of osteoblasts using histological analysis. To localize osteoblasts affected by Ra-223 treatment, transgenic C57BL/6 mice harboring 2.3-kb Col1α1 promoter-driven green fluorescence protein (GFP) was used. In Col-GFP/B6 mice, the proximal 2.3 kb Col1α1 promoter directs osteoblast-specific expression of GFP. In the femurs of Col-GFP/B6 mice, GFP-expressing osteoblasts were mainly found in the growth plate (GP) and trabeculae (Tb) in the metaphysis, with much fewer GFP-expressing osteoblasts in the diaphysis (Figure 2A). Representative images of the growth plate (GP) and trabecular regions (Tb1-3) of a Col-GFP/B6 mouse are shown in Figure 2B. Col-GFP/B6 mice were treated with or without a single dose (300 kBq/kg) of Ra-223, and the femurs were analyzed at 1, 4, or 16 weeks after Ra-223 treatment. Ra-223 treatment showed a trend towards reduction in the number of GFP-labeled osteoblasts at 1 week after treatment (Figure 2A–C). However, it did not reach statistical significance due to low sample size (*n* = 2). At 4 weeks after treatment, the decrease in osteoblast number was mainly observed in the metaphysis of the femur, an area of active trabecular bone formation (Figure 2D–F). Quantification of GFP levels by ImageJ software showed that Ra-223 significantly inhibited osteoblasts present in trabeculae (Tb1 and Tb2) but not in the growth plate region (GP) in male mice (Figure 2F). Similar results were obtained in Col-GFP/B6 mice treated with Ra-223 for 16 weeks (Figure 3). These data suggest that in normal mice, Ra-223 affects osteoblasts mainly located in the trabecular area and, to a lesser extent, in the growth plate. 

### 3.3. Ra-223 Decreases Bone Mineral Density of Col-GFP Mice

**Figure 4:**
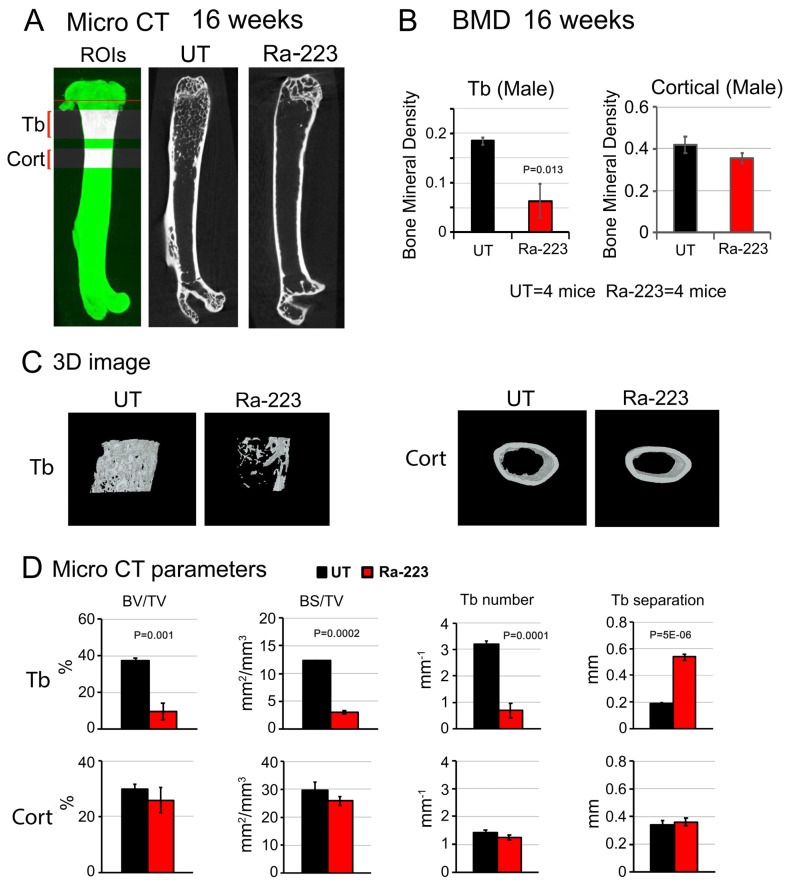
Ra-223 treatment significantly impairs trabecular bone. Non-decalcified male Col-GFP/B6 mouse femurs untreated (UT) or treated with Ra-223 for 16 weeks were analyzed by SkyScan μCT. (**A**) The scanned images of representative femurs of untreated and Ra-223-treated groups. ROIs defined the trabecular (Tb) and the cortical (Cort) regions for bone microstructure analyses. (**B**) Bone mineral density (BMD) of trabecular (Tb) and cortical (Cort) bone were determined by μCT. With a sample size of 4 mice for untreated (UT) and 4 mice in Ra-223 treated group, the effect sizes were 2.48 and 1.00 for Tb and Cort, respectively. The powers of the test for Tb and Cort were 0.93 and 0.35, respectively, using a sample *t*-test (1-sided) and 5% significance level. Ra-223 treatment for 16 weeks caused a significant decrease in bone mineral density (BMD) of trabecular but not cortical bone. (**C**) The 3D images of trabeculae and cortical bone with and without Ra-223 treatment for 16 weeks. (**D**) Bone parameters in Tb and Cort areas were determined by micro-CT. With a sample size of 4 mice for untreated (UT) and 4 mice in Ra-223 treated group, the effect sizes were 4.18, 15.86, 6.46, 9.96 for BV/TV, BS/TV, Tb number, and Tb separation, respectively. The powers of the test for BV/TV, BS/TV, Tb number, and Tb separation were 1.00 for all four parameters, using a sample *t*-test (1-sided) and 5% significance level. BV/TV, BS/TV, and Tb number were significantly decreased, and Tb separation was significantly increased by Ra-223 treatment when compared to untreated mice. However, these parameters were not significantly changed by Ra-223 in the cortical region.

To examine the effect of Ra-223 on bone microarchitecture, high-resolution micro-computed tomography (μCT) analyses of non-decalcified femurs from Col-GFP mice treated with or without Ra-223 were performed. There were significant decreases in the bone mineral density in the trabecular but not cortical bone in male mice at 16 weeks after Ra-223 treatment (Figure 4A–C). Furthermore, μCT analysis revealed a significant decrease in trabecular bone volume (BV/TV), trabecular bone surface (BS/TV), and trabecular number, and a significant increase in trabecular bone separation, in femurs of Ra-223-treated mice compared to those from untreated mice (Figure 4D). Similar results were observed in female mice at 4 weeks (Figure S3) and 16 weeks (Figure S4) after Ra-223 treatment. These observations suggest that Ra-223 decreases bone mineral density in the trabecular areas where a decrease in osteoblasts was observed. 

### 3.4. Ra-223 Decreases Tumor-Induced Osteoblasts

Next, the effects of Ra-223 on osteoblasts induced by prostate cancer cells were examined. Prostate cancer bone metastasis is mainly osteoblastic [26,27,28]. Although a few patient-derived xenografts (PDXs) have been able to recapitulate such pathological events in bone [29,30], they would need to use immune-deficient mice. Transgenic adenocarcinoma of the mouse prostate (TRAMP) is a tumor model in the immunocompetent C57BL/6 mouse [31]. Several cell lines (TRAMP-C1, -C2, and -C3) have been generated from prostate tumors from TRAMP mice [23]. TRAMP-C2 has been used for generating tumors in C57BL/6 mice. However, injecting TRAMP-C2 into C57BL/6 mouse femurs generates mainly osteolytic lesions. We have previously shown that tumor-induced bone formation is due to tumor-secreted BMP4 [2]. Thus, to promote tumor-induced bone formation, the TRAMP-C2 cell line that expressed BMP4 (TRAMP-BMP4) was generated [4,32]. This cell line was used to create a mouse model of prostate tumor-induced osteoblasts.

**Figure 5:**
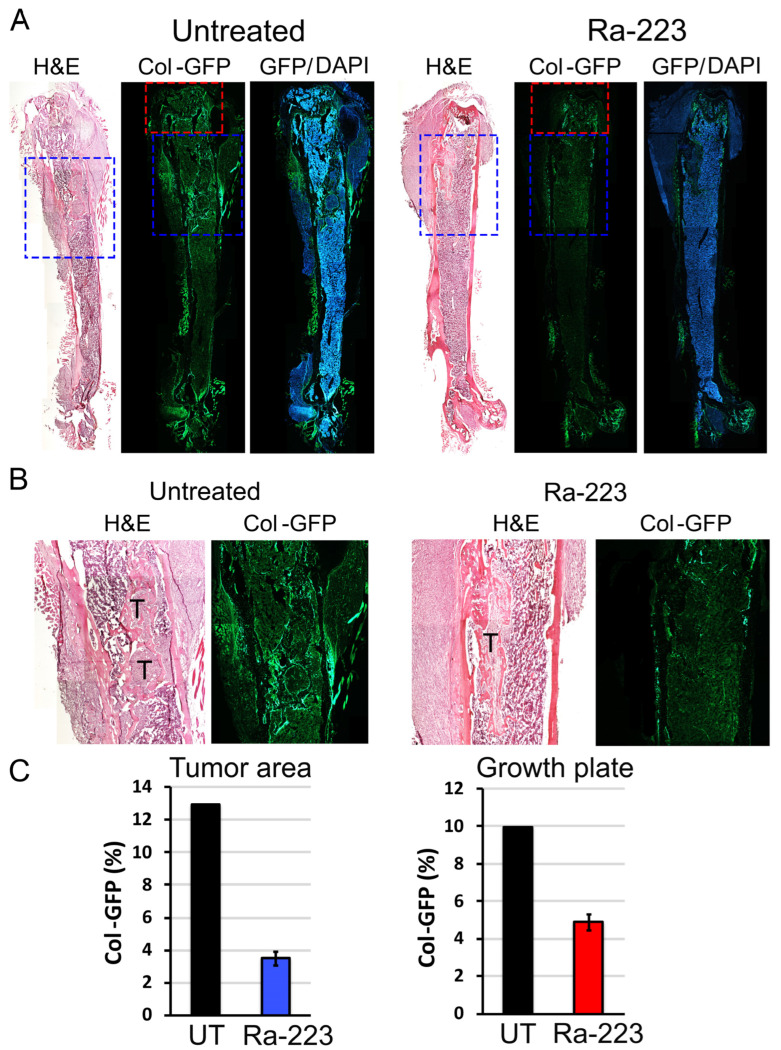
TRAMP-BMP4 tumor cells induce ectopic bone formation in the femurs of Col-GFP osteoblast reporter mice. (**A**) Histologic analysis by H&E staining and GFP of nearby sections. Blue boxes: areas with tumor and tumor-induced bone. Red boxes: growth-plate areas. (**B**) H&E staining and GFP of tumor and tumor-induced areas within the bone marrow. T: tumor. (**C**) Quantification of GFP intensity in the tumor (blue box in A) and growth plate (red box in A) areas with or without Ra-223 treatment.

TRAMP-BMP4 cells were injected into the femurs of Col-GFP mice, and the tumors were grown for 3 weeks. The mice were then treated with or without one dose of Ra-223, and mouse femurs were examined 2 weeks after treatment. Upon histological analysis of femurs, TRAMP-BMP4 cells were found to induce aberrant bone formation surrounding the tumors in the bone marrow (Figure 5A,B). The osteoblasts rimming the tumor-induced bone were GFP positive (Figure 5A,B). Ra-223 treatment led to a decrease in the GFP positive cells rimming the tumor-induced bone compared with those in untreated tumors (Figure 5C). Longer Ra-223 treatment was not performed due to tumor growth that necessitated termination of the study. Due to a low tumor uptake rate (20–40%) of TRAMP-BMP4 cells in Col-GFP mouse femurs, only a limited number of TRAMP-BMP4 tumor-bearing bones were analyzed. Regardless, these observations confirmed the previous report by Suominen et al. [21] that Ra-223 treatment significantly reduced tumor-induced osteoblasts at 2 weeks after treatment. 

## 4. Discussion

Ra-223 is being used for the treatment of bone-forming, bone metastasis of prostate cancer due to its affinity for hydroxyapatite that is enriched in tumor-induced bone. However, Ra-223 can also bind to non-tumor-affected bone and may have effects on bone health. Using osteoblast reporter mice to monitor the levels of osteoblasts in vivo, a single dose of Ra-223 led to a rapid and sustained reduction of resident osteoblasts. Because Ra-223 has a half-life of 11.4 days and bone mass is maintained at a constant level in vertebrates through bone remodeling, it is expected that osteoblasts would be replenished, and bone mass maintained after radioactivity decay. Surprisingly, recovery of luciferase activity for the duration of the 16-week follow-up time was not observed. Ra-223 was found to mainly target the osteoblasts in the trabecular rather than the cortical bone, causing a decrease in trabecular bone mineral density and bone volume that persisted at least 16 weeks after Ra-223 treatment. These data indicate that Ra-223 treatment has long-lasting effects on normal bone homeostasis, aside from its effects on tumor-induced osteoblasts. This study thus provides insights into the effect of Ra-223 beyond tumor-affected bone and suggests that preventing the effect of Ra-223 on bone health in non-tumor-affected areas will be important for avoiding the incidence of bone fractures and thus improving the quality of life for patients undergoing Ra-223 treatment. 

The observation that resident osteoblasts were not replenished after radioactivity decay is unexpected. Skeletal tissue is in a constant state of remodeling, and bone mass is maintained when the activities of osteoblasts, osteoclasts, and osteocytes are in equilibrium [33]. Because bone resorption is followed by bone formation and osteoblast progenitors secrete a factor required for osteoclast differentiation, it is assumed that the two processes are mechanistically coupled. Interestingly, using an inducible osteoblast-ablation mouse model, Corral et al. [34] demonstrated that arresting bone formation did not affect bone resorption; osteoclast activity remained the same. This continued bone resorption without bone formation resulted in bone loss, which could be prevented by an antiresorptive agent [34]. Their studies established the concept that bone formation and bone resorption are separate processes. Similar to the results of Corral et al. [34], a recovery of osteoblast-specific luciferase expression was not observed after Ra-223 treatment throughout the duration of the 16-week follow-up, bone mass was not maintained, as a decrease in trabecular bone mineral density and bone volume was observed. These observations support the notion that bone formation and bone resorption are separate processes. Although not measured in the current study, the osteoclasts likely remained active and contributed to the decrease in trabecular bone mineral density and bone volume after Ra-223-mediated osteoblast eradication. Thus, results from this study suggest that Ra-223 has damaging effects on the bone health in non-tumor-affected areas and that inhibition of osteoclast activity by antiresorptive agents may prevent the Ra-223-induced decrease in bone mineral density.

While recovery of luciferase activity after Ra-223 treatment was not observed in this study, it is possible that in pathological conditions, bone-forming activity may persist due to continued stimulation from prostate cancer cells. Thus, frequent applications of Ra-223 may be necessary to control osteoblastic bone lesions. In this study, Ra-223 at a dose of 300 kBq/kg was used. This dose was previously selected for mouse models of breast cancer studies [35] and was subsequently used in several prostate cancer bone metastasis studies [19,20,21]. Ra-223 binds to sites of active mineralization and will emit its alpha particles within a 100 µm radius (around 2–10 cell diameter), killing all cells in that region. In an osteolytic breast cancer bone metastasis model, Suominen et al. [35] reported that osteoclasts as well as tumor cells were targeted by Ra-223 based on histologic analysis. Thus, Ra-223 not only kills osteoblasts but also damages other types of cells that are close to bone surface and within the track length of Ra-223. 

The observation that Ra-223 principally targets osteoblasts in trabecular bone adjacent to growth plates is consistent with the distribution of Ra-223 reported by Abou et al. [36]; they showed that Ra-223 uptake occurred in the ossifying surfaces adjacent to the epiphyseal growth plate. Abou et al. [36] also showed that Ra-223 did not bind to tumor. This is consistent with the clinical observation that Ra-223 treatment has negligible effect on PSA responses [16]. Thus, combining Ra-223 with therapies that principally target prostate cancer cells may enhance therapy outcomes. However, the phase 3 clinical trial combining Ra-223 with abiraterone, which targets androgen-sensitive prostate cancer cells, showed an increase in bone fractures, and the majority of these occurred at skeletal sites with no bone metastasis [18]. The most common type of fracture was osteoporotic [18]. Because androgen-deprivation therapy has been shown to decrease bone mineral density [37], the current study suggests that Ra-223-mediated osteoblast depletion and decrease in bone density in non-tumor-involved bone further weakens bone structure. Consistent with the proposition that inhibition of osteoclast activity may prevent the decrease in bone mineral density from Ra-223, Trieu et al. [38] reported that the incidence of bone fractures from Ra-223 and abiraterone combination therapy could be prevented with the routine use of bone-health agents, e.g., bisphosphonates or RANKL inhibitors.

## 5. Conclusions

This study shows that Ra-223 affects the structure of non-tumor-associated bones in bone metastasis. This knowledge on the effect of Ra-223 on non-tumor-involved bone will be important for future development of Ra-223 combination therapies for treatment of patients with bmCRPC.

## Data Availability

Data will be available to interested researchers upon request.

## Supplementary Materials

The following supporting information can be downloaded at: https://www.mdpi.com/article/10.3390/cancers16142603/s1, Figure S1. Ra-223 treatment decreases luciferase activity in the bones of mouse feet. Col-Luc/SCID mice were treated with 300 kBq/kg Ra-223, and the luciferase activity was monitored for 18 weeks using an IVIS 200 imaging system. (A) Photon counts of the feet of three Col-Luc/SCID mice right before Ra-223 injection (day 0) and on days 2 and 7. (B) Photon counts of mouse feet from 0 to 18 weeks. Figure S2. Ra-223 treatment decreases tail luciferase activity in female Col-Luc/B6 osteoblast reporter mice. Five female Col-Luc/B6 reporter mice were treated with a single dose of Ra-223 and monitored for 18 weeks. (A) BLI of mice. (B) Quantitation of tail photon counts over 18 weeks. Figure S3. Ra-223 significantly decreased trabecular bone densities in female Col-GFP/B6 reporter mice 4 weeks after Ra-223 treatment. Eight 10-week-old female Col-GFP/B6 mice were used. Four mice were treated with one dose of Ra-223 at 300 kBq/kg, and the other four mice remained untreated (UT). Four weeks later, femurs of the mice were processed for micro-computed tomography (µCT) analysis. (A) Representative µCT image. (B) Bone mineral density (BMD) of trabecular (Tb) and cortical (Cort) bone as determined by µCT. With a sample size of 4 mice for untreated (UT) and 4 mice in Ra-223 treated group, the effect sizes (Cohen’s d) were 5.74 and 0.33 for Tb and Cort, respectively. The powers of the test for Tb and Cort were 1.00 and 0.11, respectively, using a sample t-test (1-sided) and 5% significance level. Ra-223 treatment for 4 weeks caused a significant decrease in bone mineral density (BMD) of trabecular but not cortical bone. (C) Representative 3-D images of trabecular and cortical bone areas of femurs. (D) Bone parameters in Tb and Cort areas were determined by micro-CT. With a sample size of 4 mice for untreated (UT) and 4 mice in Ra-223 treated group, the effect sizes were 6.32, 6.42, 5.36, 5.60 for BV/TV, BS/TV, Tb number, and Tb separation, respectively. The powers of the test for BV/TV, BS/TV, Tb number, and Tb separation were 1.00 for all four parameters, using a sample t-test (1-sided) and 5% significance level. Figure S4. Ra-223 significantly decreased trabecular bone in female Col-GFP/B6 reporter mice 16 weeks after treatment. Eight 10-week-old female Col-GFP/B6 reporter mice were studied. Four mice were treated with one dose of Ra-223 at 300 kBq/kg, and the other four mice remained untreated (UT). Sixteen weeks later, mouse femurs were processed for µCT analysis. (A) Representative µCT image. (B) Bone mineral densities of trabecular (Tb) and cortical (Cort) bone as determined by µCT. With a sample size of 4 mice for untreated (UT) and 4 mice in Ra-223 treated group, the effect sizes (Cohen’s d) were 3.59 and 0.09 for Tb and Cort, respectively. The powers of the test for Tb and Cort were 0.99 and 0.06, respectively, using a sample *t*-test (1-sided) and 5% significance level. Ra-223 treatment for 16 weeks caused a significant decrease in bone mineral density (BMD) of trabecular but not cortical bone. (C) Representative 3-D images of trabecular and cortical bone areas of femurs. (D) Bone parameters in Tb and Cort areas were determined by micro-CT. With a sample size of 4 mice for untreated (UT) and 4 mice in Ra-223 treated group, the effect sizes were 5.96, 6.46, 7.20, 10.25 for BV/TV, BS/TV, Tb number, and Tb separation, respectively. The powers of the test for BV/TV, BS/TV, Tb number, and Tb separation were 1.00 for all four parameters, using a sample t-test (1-sided) and 5% significance level.
