# Supplementary material for: Radium-223 Treatment Produces Prolonged Suppression of Resident Osteoblasts and Decreased Bone Mineral Density in Trabecular Bone in Osteoblast Reporter Mice
Source: Cancers (Basel). 2024 Jul 21;16(14):2603. doi: 10.3390/cancers16142603 (PMC11274981; doi:10.3390/cancers16142603)
Supplement: Supplementary file 1 [file cancers-16-02603-s001.zip › Supplementary Figures.pdf]

Supplementary Figures and Figure Legends

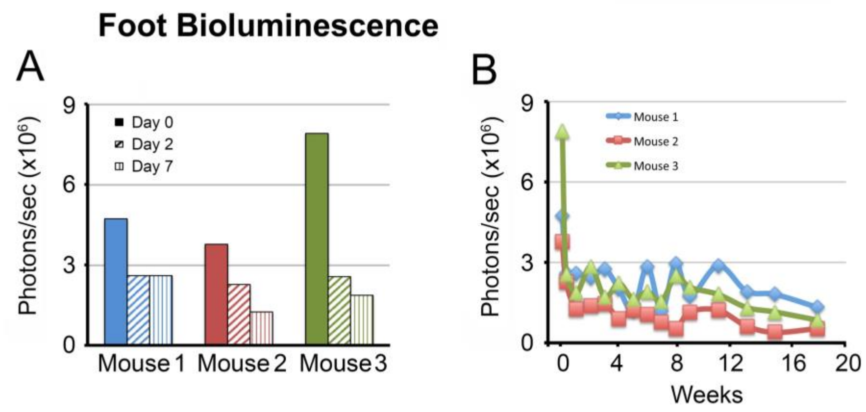

Figure S1. Ra-223 treatment decreases luciferase activity in the bones of mouse feet. Col-Luc/SCID mice were treated with 300 kBq/kg Ra-223, and the luciferase activity was monitored for 18 weeks using an IVIS 200 imaging system. (A) Photon counts of the feet of three Col-Luc/SCID mice right before Ra-223 injection (day 0) and on days 2 and 7. (B) Photon counts of mouse feet from 0 to 18 weeks.

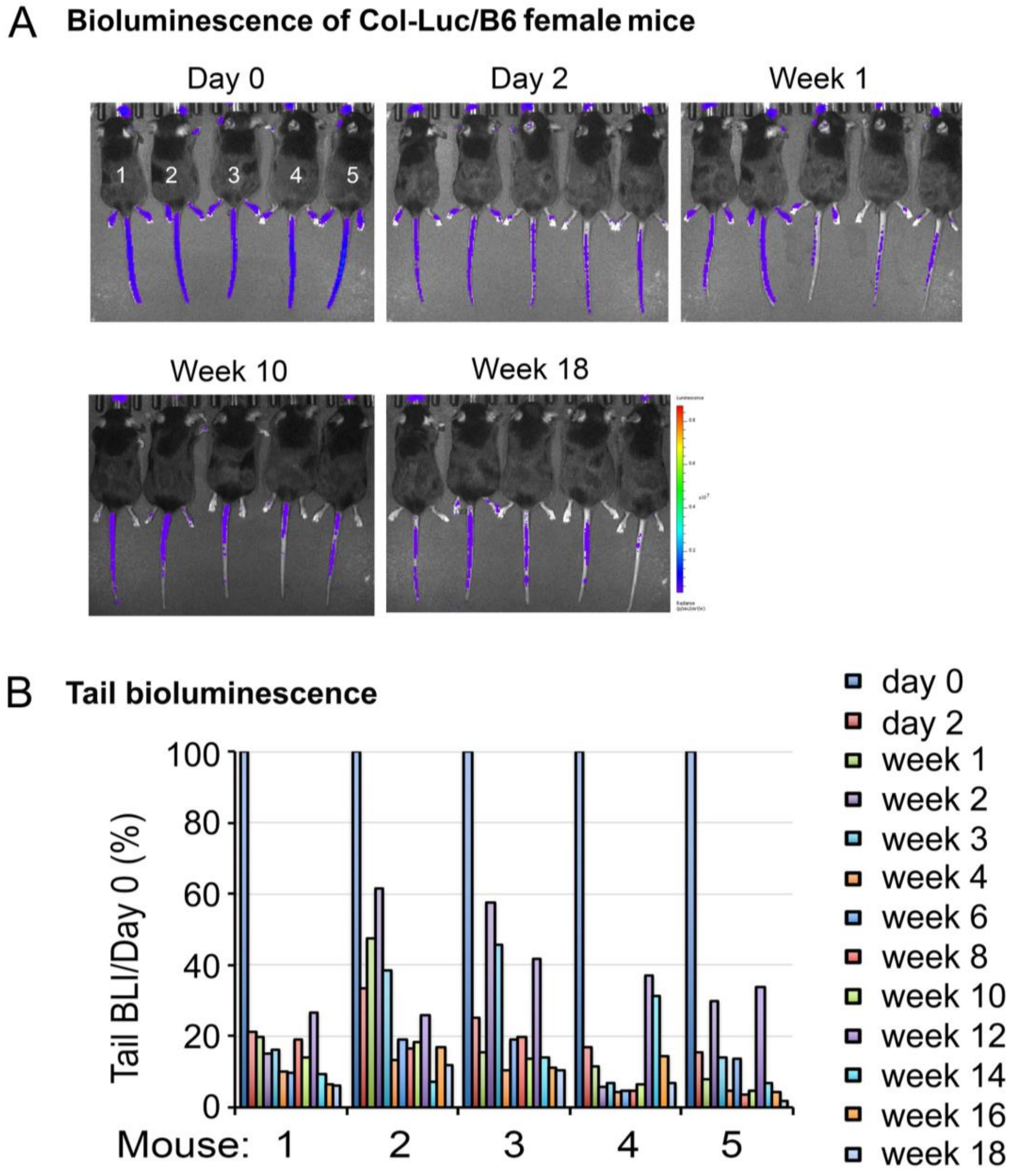

Figure S2. Ra-223 treatment decreases tail luciferase activity in female Col-Luc/B6 osteoblast reporter mice. Five female Col-Luc/B6 reporter mice were treated with a single dose of Ra-223 and monitored for 18 weeks. (A) BLI of mice. (B) Quantitation of tail photon counts over 18 weeks.

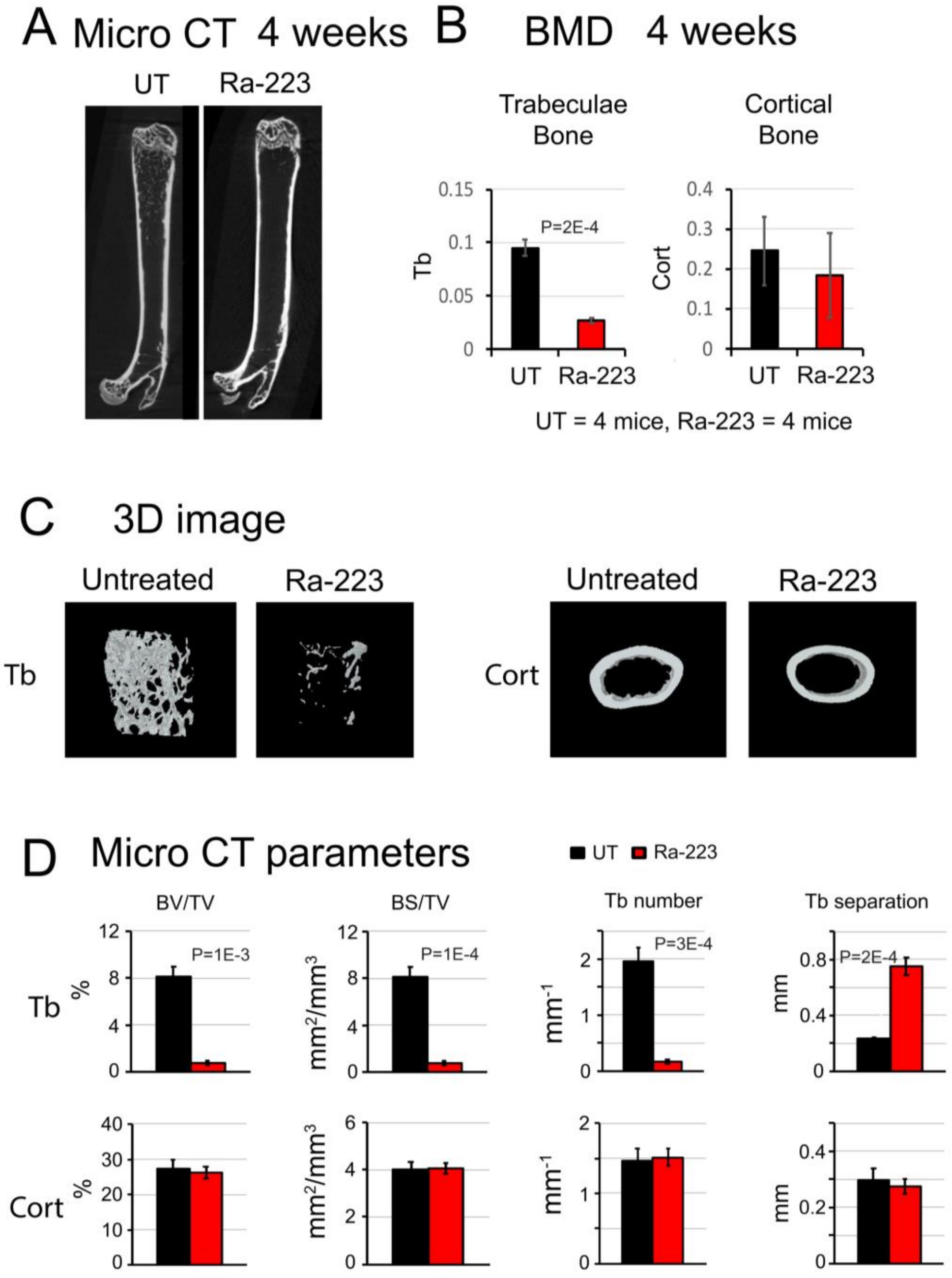

Figure S3. Ra-223 significantly decreased trabecular bone densities in female Col-GFP/B6 reporter mice 4 weeks after Ra-223 treatment. Eight 10-week-old female Col-GFP/B6 mice were used. Four mice were treated with one dose of Ra-223 at 300 kBq/kg, and the other four mice remained untreated (UT). Four weeks later, femurs of the mice were processed for micro-computed tomography (μCT) analysis. (A) Representative μCT image. (B) Bone mineral density (BMD) of trabecular (Tb) and cortical (Cort) bone as determined by μCT. With a sample size of 4 mice for untreated (UT) and 4 mice in Ra-223 treated group, the effect sizes (Cohen's *d*) were 5.74 and 0.33 for Tb and Cort, respectively. The powers of the test for Tb and Cort were 1.00 and 0.11, respectively, using a sample

t-test (1-sided) and 5% significance level. Ra-223 treatment for 4 weeks caused a significant decrease in bone mineral density (BMD) of trabecular but not cortical bone. (C) Representative 3-D images of trabecular and cortical bone areas of femurs. (D) Bone parameters in Tb and Cort areas were determined by micro CT. With a sample size of 4 mice for untreated (UT) and 4 mice in Ra-223 treated group, the effect sizes were 6.32, 6.42, 5.36, 5.60 for BV/TV, BS/TV, Tb number, and Tb separation, respectively. The powers of the test for BV/TV, BS/TV, Tb number, and Tb separation were 1.00 for all four parameters, using a sample *t*-test (1-sided) and 5% significance level.

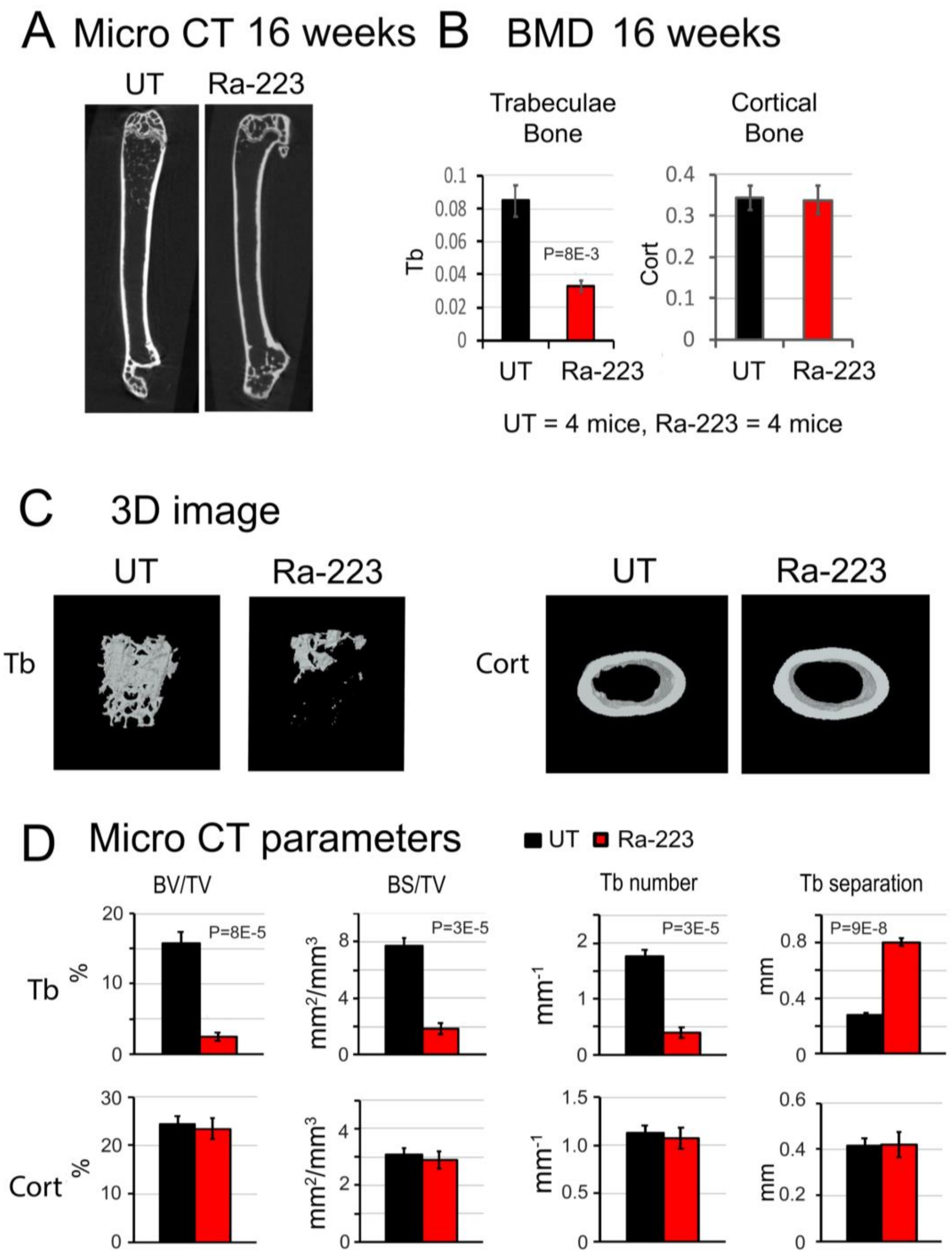

Figure S4. Ra-223 significantly decreased trabecular bone in female Col-GFP/B6 reporter mice 16 weeks after treatment. Eight 10-week-old female Col-GFP/B6 reporter mice were studied. Four mice were treated with one dose of Ra-223 at 300 kBq/kg, and the other four mice remained untreated (UT). Sixteen weeks later, mouse femurs were processed for μCT analysis. (A) Representative μCT image. (B) Bone mineral densities of trabecular (Tb) and cortical (Cort) bone as determined by μCT. With a sample size of 4 mice for untreated (UT) and 4 mice in Ra-223 treated group, the effect sizes

(Cohen's *d*) were 3.59 and 0.09 for Tb and Cort, respectively. The powers of the test for Tb and Cort were 0.99 and 0.06, respectively, using a sample *t*-test (1-sided) and 5% significance level. Ra-223 treatment for 16 weeks caused a significant decrease in bone mineral density (BMD) of trabecular but not cortical bone. (C) Representative 3-D images of trabecular and cortical bone areas of femurs. (D) Bone parameters in Tb and Cort areas were determined by micro CT. With a sample size of 4 mice for untreated (UT) and 4 mice in Ra-223 treated group, the effect sizes were 5.96, 6.46, 7.20, 10.25 for BV/TV, BS/TV, Tb number, and Tb separation, respectively. The powers of the test for BV/TV, BS/TV, Tb number, and Tb separation were 1.00 for all four parameters, using a sample *t*-test (1-sided) and 5% significance level.
